# Gamma-hydroxybutyric acid-induced organic delirium complicated by polydrug use successfully treated with electroconvulsive therapy: a case report

**DOI:** 10.1186/s13256-021-03182-w

**Published:** 2021-12-16

**Authors:** Mads F. Kjærgaard, Poul Videbech, Jens J. Nørbæk, Bjørn H. Ebdrup

**Affiliations:** 1 Mental Health Centre Glostrup, University of Copenhagen, Glostrup, Denmark; 2 Center for Neuropsychiatric Depression Research, CNSR & Centre for Clinical Intervention and Neuropsychiatric Schizophrenia Research, CINS, Mental Health Centre Glostrup, University of Copenhagen, Nordstjernevej 41, DK-2600 Glostrup, Denmark; 3 Center for Clinical Intervention and Neuropsychiatric Schizophrenia Research, CINS, and Center for Neuropsychiatric Schizophrenia Research, CNSR, Mental Health Centre Glostrup, University of Copenhagen, Glostrup, Denmark; 4 Department of Clinical Medicine, Faculty of Health and Medical Sciences, University of Copenhagen, Copenhagen, Denmark

**Keywords:** Electroconvulsive therapy (ECT), Gamma-hydroxybutyric acid (GHB), Withdrawal, Delirium, Intensive care unit (ICU)

## Abstract

**Background:**

Patients with gamma-hydroxybutyric acid withdrawal symptoms are at high risk of developing organic delirium, which can be fatal. The recommended first-line treatment is benzodiazepines, but treatment-resistant cases are frequent. Here we describe a case of successful bilateral electroconvulsive therapy in a patient with severe and highly agitated acute organic delirium induced by gamma-hydroxybutyric acid withdrawal and complicated by polydrug use resistant to first-line treatment. To our knowledge, this is the first report on the effect of electroconvulsive therapy on treatment-resistant delirium caused by gamma-hydroxybutyric acid withdrawal.

**Case presentation:**

A 21-year-old Danish man diagnosed with untreated attention deficit hyperactivity disorder developed severely agitated acute organic delirium caused by gamma-hydroxybutyric acid withdrawal in a Danish psychiatric ward. The patient was subjected to physical restraints and transferred to the intensive care unit for treatment. During the next 10 days, the patient showed no clinical improvement despite first-line, high-dose benzodiazepines along with intense supportive treatment with propofol, phenobarbital, and antipsychotics. On day 11, bilateral frontotemporal electroconvulsive therapy treatment was initiated and full clinical recovery was obtained after four sessions.

**Discussion:**

The full clinical remission after four electroconvulsive therapy sessions, strongly supports that electroconvulsive therapy may be an effective treatment when severe delirium induced by gamma-hydroxybutyric acid withdrawal is resistant to conventional first-line treatment with benzodiazepines. Moreover, this case illustrates that clinically effective seizures were achieved despite intensive concurrent exposure to anticonvulsive drugs. Therefore, this case report encourages consideration of electroconvulsive therapy in patients with gamma-hydroxybutyric acid delirium who are resistant to psychopharmacological treatment.

## Background

Gamma-hydroxybutyric acid (GHB) emerged as a dietary supplement used by bodybuilders in the 1970–1980s, and gradually became a recreational drug in the club scene [1]. The prevalence of GHB use is about 1% in the USA. GHB is highly addictive, and people with physical dependence will need rapidly increasing doses [1]. GHB intoxication resembles intoxication with sedative-type drugs such as barbiturates, benzodiazepines, or ethanol, and diagnosis and management of GHB withdrawal symptoms constitute a frequent clinical challenge [1]. Withdrawal symptoms typically develop within 24 hours of last GHB intake and include tremor, fever, diaphoresis, anxiety, agitation, insomnia, tachycardia, confusion, paranoia, hallucinations, seizure, and ultimately delirium. Delirium, or “acute organic psycho-syndrome,” is defined by the World Health Organization (WHO) International Classification of Diseases 10th Revision (ICD-10) as an “etiologically nonspecific organic cerebral syndrome characterized by concurrent disturbances of consciousness and attention, perception, thinking, memory, psychomotor behaviour, emotion, and the sleep-wake schedule.” [2] Delirium caused by GHB-withdrawal can be fatal [1].

Although GHB is a potent modulator of the gamma-aminobutyric acid (GABA)-B receptor subtype, the first-line treatment of both GHB withdrawal syndrome and GHB-induced delirium is benzodiazepines, which modulate the GABA-A receptor subtypes [1]. Alternative pharmacological approaches include barbiturates, baclofen, and experimental tapering with pharmaceutical GHB, but the evidence is limited [1, 3]. A further challenge is that up to 86% of GHB users report polydrug use [1], and polydrug use with stimulants such as cocaine or amphetamine may complicate GHB withdrawal symptoms [4].

We present a 21-year-old man with a highly agitated, acute organic delirium induced by GHB withdrawal, complicated by co-abuse of cocaine and an occult infection. The psychiatric condition was resistant to first-line benzodiazepine treatment and supporting psychopharmacological treatment at the intensive care unit (ICU). Due to the acute life-threatening condition, electroconvulsive therapy (ECT) was initiated, and after four ECT sessions the patient achieved full clinical remission. To our knowledge, this is the first report supporting that ECT may constitute a potentially novel approach in the treatment of severe delirium induced by GHB withdrawal resistant to conventional first-line treatment with benzodiazepines.

## Case presentation

A 21-year-old Danish man diagnosed with attention deficit hyperactivity disorder (ADHD) in adolescence, which was currently untreated, was involuntarily admitted to a psychiatric ward by the police in an agitated and aggressive state after a violent conflict with his parents. Clinically, the patient’s state was compatible with acute intoxication, and the patient confirmed “drug intake,” but quantification could not be specified at admission. Eight months prior, the patient had spent 2 weeks in deep propofol sedation at an intensive care unit (ICU) with acute organic delirium due to GHB withdrawal.

At the psychiatric ward, the patient’s threatening and aggressive behavior intensified. To avoid violent incidents, the patient was subjected to physical restraint (abdominal belt) and tablet lorazepam 4 mg was administered twice. The patient was disorientated, started expressing paranoid ideas, and developed diaphoresis, tachycardia (heart rate 130 beats per min), and hyperthermia (37.8 °C, tympanic). Because he started biting the metal locks on his abdominal belt, his hands and feet were also restrained.

On the second day, the patient was diagnosed with acute organic delirium caused by GHB withdrawal and treatment was initiated. In accordance with ICD-10^2^, the diagnosis was based upon the clinical presentation in combination with a history of GHB substance abuse. The patient was treated with a cumulative dose of 300 mg diazepam (40 mg as oral administration and 260 mg intravenously), without induction of sleep. To obtain sedation the restrained patient was transferred to the ICU, where he was intubated and sedated with intravenous infusions of propofol (up to 16 mg/kg/hour) and sufentanil (up to 100 μg/hour).

On days 3–6, the patient remained deeply sedated and on mechanical ventilation. To counteract potential withdrawal symptoms, clonidine 225 μg was administered every 6 hours via a nasogastric tube (NG tube), intravenous diazepam 20 mg every 5 hours, and continuous midazolam infusions (up to 1 mg/kg/hour). To prevent Wernicke encephalopathy, intravenous thiamine 200 mg and vitamin B 2 ml solution were given. Due to elevated C-reactive protein (64 mg/L) and white blood cell count (leukocytes 12.2 × 10^9^/L; neutrophils 9.1 × 10^9^/L), empiric treatment with piperacillin/tazobactam 4 g every 6 hours was initiated.

On day 6, a first wake-up call was attempted. However, upon awakening the patient was disoriented and severely agitated, and he was readily reintubated and resedated. The psychopharmacological treatment was intensified with olanzapine 20 mg/day (via NG-tube), intravenous diazepam 40 mg every 5 hours, clonidine 225 μg every 6 hours (via NG-tube), and continuous midazolam infusions (up to 1 mg/kg/hour).

On days 7–9, the intravenous diazepam was increased to 60 mg every 5 hours, and to supplement the propofol sedation, methadone 10 mg twice a day (via NG-tube) was initiated.

On day 10, a second wake-up was attempted. Again, the patient was disoriented, reported of ants crawling in the room, and presented with severe agitation. Physical restraints were reinstated and intravenous haloperidol 30 mg/day was added.

**Fig. 1:**
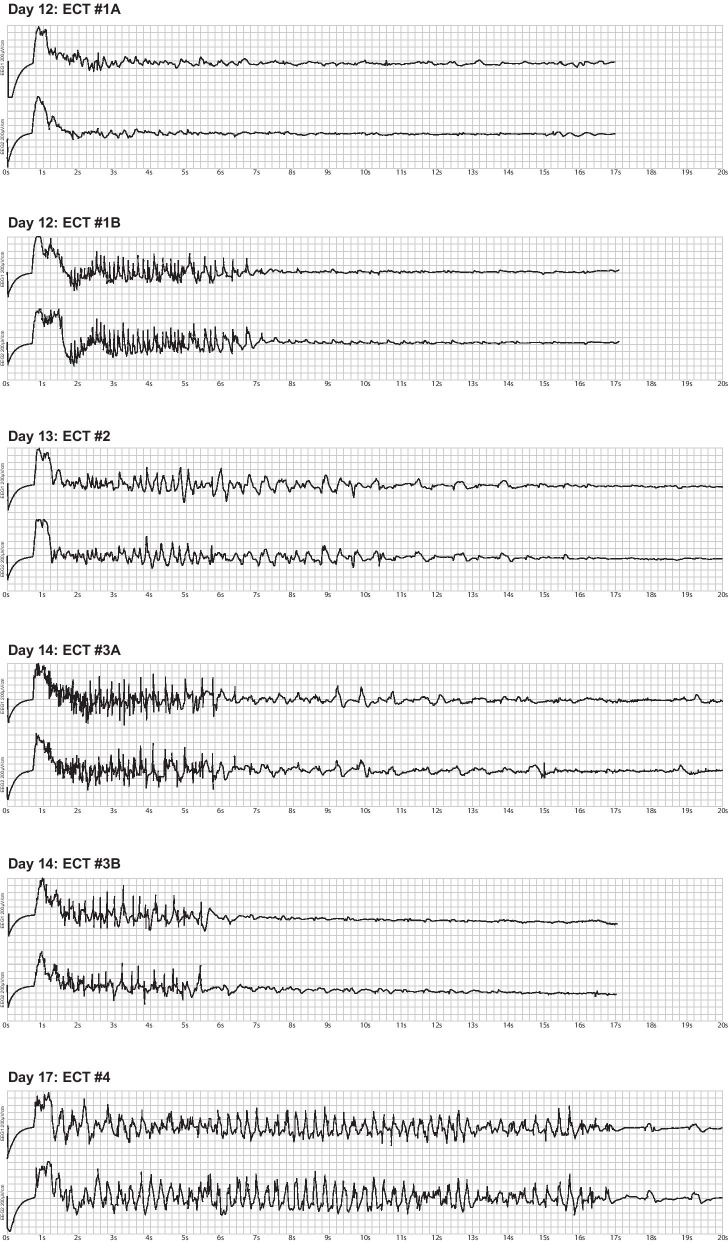
The electroconvulsive therapy read-out from the four electroconvulsive therapy treatments. All electroconvulsive therapy doses given with maximal energy of 200% (1008 mC), Thymatron System IV (SOMATICS, LLS, Venice, FL, United States). See text for details

On day 11, the psychiatrists decided to initiate bilateral frontotemporal ECT as “en bloc” treatment, that is ECT on three consecutive days. Medication status during the three en bloc ECTs was: olanzapine 20 mg twice a day (BID, via NG-tube), methadone 10 mg BID (via NG-tube), intravenous phenobarbital 100 mg every 4 hours, pregabalin 150 mg BID (via NG-tube), haloperidol 5 mg as needed (PRN), up to 50 mg a day, clonidine 225 mg every 6 hours (via NG-tube), and continuous infusions of midazolam (up to 1 mg/kg/hour). Because treatment with high doses of benzodiazepines with long half-lives could compromise seizure induction, the starting ECT dose was set at the maximal energy of 200% (1008 mC) along with intravenous flumazenil 0.4 mg. The ECT series are shown in Fig. 1.

On day 12, the first ECT was administered (ECT #1A). Due to an administrative error, flumazenil was not administered, and restimulation with concurrent flumazenil was performed (ECT #1B). After ECT, the patient remained awake although clinically unchanged.

On day 13, the second ECT was administered (ECT #2). Subsequently, the patient’s level of agitation decreased, he briefly responded adequately to verbal communication and sleep improved.

On day 14, the third ECT (ECT #3A) was administered. Due to seizure at threshold level, restimulation after hyperventilation was performed (ECT #3B). The patient was less agitated and engaged in brief conversations. Haloperidol was increased to 20 mg every 6 hours. On day 15, 50 mg hydroxyzine once a day (QD) was added.

On day 16, agitation had completely subsided, restraints were removed, and the patient cooperated with the remaining treatment.

On day 17, the fourth ECT was administered (ECT #4). On day 18, the patient displayed no neurological or psychiatric symptoms, all sedatives and psychopharmacological treatment were discontinued, and he was transferred to a general internal medicine ward.

On day 19, the patient was still in complete remission, and he insisted on discharge. Upon discharge the patient confirmed daily use of GHB and cocaine prior to hospitalization, and he provided oral and written consent to report his case in the medical literature. Since this is a case report, ethical approval was waived according to Danish regulations.

## Discussion and conclusions

The patient received first-line pharmacological treatment with high doses of benzodiazepines and supporting treatment with combinations of benzodiazepines, propofol, clonidine, olanzapine, methadone, haloperidol, phenobarbital, pregabalin, and hydroxyzine. The patient displayed no clinical improvement until after the second day of ECT, and full clinical recovery was obtained after four sessions of ECT.

The ICD-10 diagnosis of organic delirium caused by GHB withdrawal was based on the previous medical history and the characteristic clinical presentation [2]. Although the patient’s drug intake was not quantified at admission, the patient further confirmed daily use of GHB and cocaine after the delirious state had resolved. Several factors may have complicated the clinical course. In line with previous reports, the patient’s resistance to psychopharmacological treatment may have been aggravated by his concurrent cocaine abuse [4]. Moreover, infections may lead to and aggravate preexisting delirium, and infections are associated with increased morbidity, length of hospital stay, and mortality [5]. Finally, repeated withdrawal episodes of alcohol (a GABA-A receptor modulator) and potentially also withdrawal episodes from benzodiazepines are associated with more severe withdrawal symptoms, a phenomenon known as kindling. We speculate that GHB may also induce a similar kindling effect.

In Scandinavia, ECT is considered standard treatment for delirium that is resistant to pharmaceutical treatment [6]. The mechanism of action of ECT on organic delirium is unknown, but the prominent hypotheses are effects on extended cortical–thalamic–cortical signaling, a potential normalization of a dysfunctional hypothalamic–pituitary–adrenal (HPA) system, or neurotropic impact on structures in the limbic system [6]. Nevertheless, intense treatment with anticonvulsive drugs, that is benzodiazepines, pregabalin, and thiobarbiturates constitute potential barriers for seizure induction. In the current case, administration of a benzodiazepine with a short half-life (midazolam), pausing infusions in the mornings prior to ECT (at around 5 AM), administration of intravenous flumazenil 0.4 mg, and application of maximal energy (1008 mC) successfully mitigated this challenge.

Concurrent with ECT, the patient was undergoing a broad range of pharmacological treatments, thus we cannot infer that ECT is the cause of the clinical remission. However, the patient’s prior extended ICU stay in a similar condition but without ECT, the long duration of the current delirious state, and the striking improvement observed after the second ECT session accompanied by full clinical remission after four ECT sessions, strongly support that ECT may be an effective treatment when severe delirium induced by GHB withdrawal is resistant to conventional first-line treatment with benzodiazepines. Further, our case demonstrates that clinically effective seizures can be achieved despite concurrent treatment with anticonvulsive drugs. Future studies investigating the potential of ECT against delirium caused by GHB withdrawal with and without comorbid polydrug use are encouraged.

## Data Availability

Data sharing is not applicable to this article as no datasets were generated or analysed during the current study.

## Abbreviations

BID: *Bis in die* (Twice a day)
ECT: Electroconvulsive therapy
GABA: Gamma-aminobutyric acid
GHB: Gamma-hydroxybutyric acid
ICU: Intensive care unit
NG-tube: Nasogastric-tube
PRN: *Pro re nata* (As needed)
